# Skeletal muscle Ca_V_1.1 channelopathies

**DOI:** 10.1007/s00424-020-02368-3

**Published:** 2020-03-28

**Authors:** Bernhard E. Flucher

**Affiliations:** Department of Physiology and Medical Biophysics, Medical University Innsbruck, Schöpfstraße 41, A6020 Innsbruck, Austria

**Keywords:** Voltage-gated calcium channel, Skeletal muscle, Hypokalemic periodic paralysis, Myotonic dystrophy, Malignant hyperthermia susceptibility, Ca_V_1.1-myopathy, Native American myopathy

## Abstract

Ca_V_1.1 is specifically expressed in skeletal muscle where it functions as voltage sensor of skeletal muscle excitation-contraction (EC) coupling independently of its functions as L-type calcium channel. Consequently, all known Ca_V_1.1-related diseases are muscle diseases and the molecular and cellular disease mechanisms relate to the dual functions of Ca_V_1.1 in this tissue. To date, four types of muscle diseases are known that can be linked to mutations in the *CACNA1S* gene or to splicing defects. These are hypo- and normokalemic periodic paralysis, malignant hyperthermia susceptibility, Ca_V_1.1-related myopathies, and myotonic dystrophy type 1. In addition, the Ca_V_1.1 function in EC coupling is perturbed in Native American myopathy, arising from mutations in the Ca_V_1.1-associated protein STAC3. Here, we first address general considerations concerning the possible roles of Ca_V_1.1 in disease and then discuss the state of the art regarding the pathophysiology of the Ca_V_1.1-related skeletal muscle diseases with an emphasis on molecular disease mechanisms.

## Introduction

The skeletal muscle calcium channel (Ca_V_1.1) is the prototypical voltage-gated calcium channel. Ca_V_1.1 was the first of the ten voltage-gated calcium channel (Ca_V_) isoforms to be isolated from rabbit skeletal muscle [17], the first to be cloned and sequenced [87], and the first for which a high-resolution cryo-EM structure has been solved [105, 106]. Based on sequence homology, its gating properties, and its pharmacological profile, Ca_V_1.1 belongs to the class of L-type calcium channels [13]. Due to its binding and sensitivity to the dihydropyridine class of channel blockers, Ca_V_1.1 is commonly referred to as dihydropyridine (DHP) receptor.

Ca_V_1.1 is specifically expressed in skeletal muscle, and at least in adult skeletal muscle, it is the only Ca_V_ isoform expressed at relevant levels. In light of the preponderance of muscle tissue—it is the largest organ of our body—and of Ca_V_1.1’s central role in controlling muscle contraction [56, 88], any defects in its function are likely to result in disease. Nevertheless, the tally of known Ca_V_1.1 channelopathies is rather terse. Why is this so? There may be a number of reasons: First its central role in excitation-contraction (EC) coupling does not allow major defects or even loss of function. Consequently, sequence variants with severe effects on Ca_V_1.1 function would not be inheritable. Secondly, skeletal muscle is an amazingly plastic tissue, capable of compensating lesser functional aberrations [4]. Therefore, minor functional defects on the molecular or cellular level may not result in noticeably reduced motor function at all, or present pathology only in combination with additional stressors like physical exertion or during age-related sarcopenia. Thirdly, in mature skeletal muscle, Ca_V_1.1 does not function as calcium channel. Therefore, any defects resulting primarily in alterations or even loss of channel function might have little to no effect on its primary physiological function in skeletal muscle EC coupling. Nevertheless, the existing Ca_V_1.1-related diseases are of exceptional interest in that they provide exciting insights in molecular mechanisms of Ca_V_1.1 function as well as in diverse and unusual patho-mechanisms.

## Ca_V_1.1’s role in skeletal muscle excitation-contraction coupling

Ca_V_1.1 is the voltage sensor for skeletal muscle EC coupling [35]. Similar to the role of Ca_V_1.2 in heart and smooth muscle, Ca_V_1.1 senses the depolarization of the muscle cell during bursts of action potentials and leads to a rapid increase of the myoplasmic free calcium concentration, which in turn triggers shortening of the contractile elements and force production. Also, like Ca_V_1.2, it does so by a functional interaction with the calcium release channel in the sarcoplasmic reticulum (SR), called the ryanodine receptor (RyR), which is a major source of the calcium transient in all muscles. However, unlike the interaction between Ca_V_1.2 and RyR2 in cardiac myocytes that depends on calcium-induced calcium release, in skeletal muscle, Ca_V_1.1 and RyR1 interact physically with each other and this interaction is independent of the influx of calcium through the Ca_V_1.1 channel [27]. In fact, in a landmark experiment frog muscle was shown to continue twitching for about 20 min in a bath solution containing high concentrations of calcium chelators, thus establishing the independence of skeletal muscle EC coupling from extracellular calcium [2]. Therefore, whereas in the heart EC coupling and consequently the force of myocardial contraction strongly depends on the magnitude and properties of the L-type calcium current, in skeletal muscle, this is not the case. Consequently, deficiencies or even the loss of Ca_V_1.1 channel functions will not affect its primary function in skeletal muscle EC coupling.

While the channel function of Ca_V_1.1 is dispensable, its voltage-sensing function is essential for skeletal muscle contraction. The spontaneous null-mutant of *Cacna1S*, the gene encoding Ca_V_1.1, in *dysgenic* mice results in completely paralyzed muscles and in the death of the mice at birth from respiratory failure [88]. Dysgenic myotubes lack depolarization-induced calcium transients, even though they are electrically active and the RyR1 is expressed and functional, as demonstrated by the presence of caffeine-induced calcium transients [74]. Depolarization-induced calcium transients and contractility can be restored in *dysgenic* myotubes by reconstitution with recombinant Ca_V_1.1 [88]. Thus, Ca_V_1.1 couples membrane depolarization to calcium release from the intracellular stores, distinguishing it as the voltage sensor for skeletal muscle EC coupling.

**Fig. 1:**
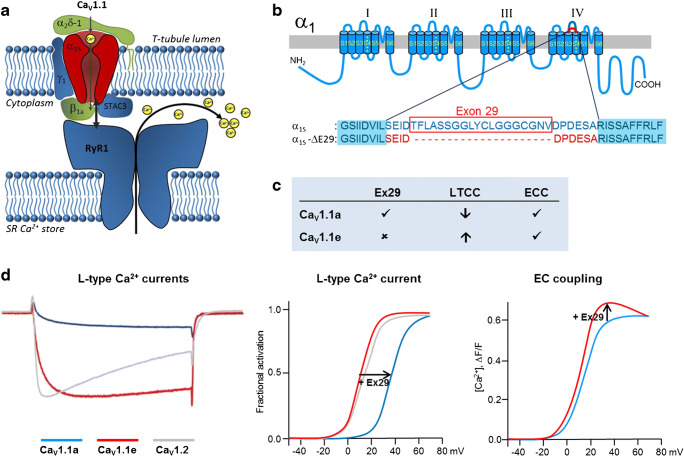
Functions of Ca_V_1.1 as L-type calcium channel and voltage sensor in EC coupling in skeletal muscle. **a** In skeletal muscle, EC coupling Ca_V_1.1 functions as voltage sensor and activates SR calcium release by interacting with RyR1 (directly or mediated by associated proteins like STAC3). **b** Domain structure of Ca_V_1.1 highlighting the alternatively spliced exon 29. **c** The two splice variants differ in their function as calcium channel but not in EC coupling. **d** Comparison of calcium currents of Ca_V_1.1a (with exon 29; blue) and Ca_V_1.1e (without exon 29; red) and Ca_V_1.2 (gray). The voltage-dependence curves show that inclusion of exon 29 right-shifts V½ of current activation but not that of EC coupling. Note that the augmented calcium influx in Ca_V_1.1e adds an extra (Cd/La-dependent) component to the depolarization-dependent calcium signals [96]

This physiological role requires functional voltage-sensing domains (although not necessarily all four of them) and a mechanism to physically couple the voltage sensor motion to activation of the RyR1 calcium release channel. Seminal freeze-fracture electron microscopy studies have demonstrated that in junctional T-tubules and plasma membrane-SR junctions, Ca_V_1.1s are organized in groups of four (called tetrads) directly opposite the cytoplasmic “foot”-domains of the RyR1 homo-tetramer and that this striking organization is isoform-specific for both Ca_V_1.1 and RyR1 [5, 27]. Moreover, sequences in Ca_V_1.1 have been identified that are essential for both its organization in tetrads and its functional interaction with the RyR1 [31, 45, 86]. Together, these findings strongly support a mechanical EC coupling model via protein-protein interactions (Fig. 1).

Allosteric interactions between Ca_V_1.1 and RyR1 activate the opening of the SR release channel in response to Ca_V_1.1 voltage sensor activation. However, to date, it is still debated whether this interaction is direct or indirectly mediated by additional components of the macromolecular EC coupling complex [75]. With regard to Ca_V_1.1-related pathology, this functional interaction with the RyR1 and possibly further proteins suggests that any mutations in Ca_V_1.1 occluding the interaction with RyR1 will result in failure of EC coupling and consequently in death. On the other hand, mutations that modulate Ca_V_1.1’s interaction with RyR1 will likely present a phenotype like that of RyR1 mutations itself. If additional proteins significantly participate in the functional Ca_V_1.1-RyR1 coupling, the prediction is that these too are candidates for EC coupling disease genes with similar phenotypes as in RyR1 or Ca_V_1.1 mutations.

High-voltage activated Ca_V_ channels typically exist as multi-subunit complexes comprising the pore-forming α_1_ subunit and an auxiliary extracellular α_2_δ and a cytoplasmic β subunit [12]. In skeletal muscle, the complex specifically contains Ca_V_1.1 (α_1S_), α_2_δ-1, β_1a_, and the γ_1_ subunit. The α_2_δ-1 subunit shapes the typical slow activation kinetics of skeletal muscle L-type calcium currents but has no known effects on EC coupling [66, 67]. α_2_δ-1 knockout mice are viable and show no apparent motor defects [28]. In contrast, the β_1a_ subunit is essential for skeletal muscle EC coupling; its knockout in mice results in paralyzed muscles and perinatal death, a phenotype similar to that of the *dysgenic* (Ca_V_1.1-null) mice [32]. Studies in myotubes from mice and zebrafish have shown that β_1a_ is important for the organization of Ca_V_1.1 in tetrads opposite RyR1 and for the voltage-sensing function of Ca_V_1.1 [18, 77, 78]. Thus, β_1a_ is the third essential component of the EC coupling complex and a role in coupling the voltage sensor to the release channel has been proposed [16]. The transmembrane γ_1_ subunit is not essential for muscle function, as γ_1_ knockout mice are viable and normal [99]. However, the γ_1_ subunit modulates the voltage dependence of inactivation of both Ca_V_1.1 L-type currents and EC coupling [1, 100]. Thus, of the classical auxiliary subunits of the calcium channel complex, only the β_1a_ subunit would be a candidate for an EC coupling disease gene. Nevertheless, to our knowledge, no *CACNB1* variants have been associated with skeletal muscle disease.

Recently, another essential EC coupling protein has been discovered. STAC3 (SH3 and cysteine-rich domain 3) is one of three members of a family of scaffold proteins containing a C1 and tandem SH3 domains. The STAC3 isoform is specifically and exclusively expressed in skeletal muscle where it colocalizes with Ca_V_1.1 and RyR1 in the triad junctions [23]. Knockout of STAC3 in mice and fish results in a failure of EC coupling, while muscle excitability and caffeine-induced SR calcium release remain intact [36, 63]. In heterologous cells, STAC3 is critical for efficient functional expression of Ca_V_1.1 channels, suggesting a chaperone function for membrane expression [71].

Two distinct interactions of STAC3 with Ca_V_1.1 have been demonstrated. A specific interaction with the C-terminus of Ca_V_1 channels promotes the incorporation of STAC3 into skeletal muscle triads and possibly interferes with the calmodulin-mediated calcium-dependent inactivation of L-type calcium currents [8, 9]. A specific interaction with the critical EC coupling sequence in the Ca_V_1.1 II–III loop is important for proper EC coupling [73, 103]. Thus, in the skeletal muscle calcium channel complex, STAC3 may play multiple roles as chaperone for Ca_V_1.1, as modulator of current properties, and as essential component in the mechanism coupling Ca_V_1.1 to RyR1, or even the coupling protein proper [23]. Considering these properties, STAC3 can be rightly viewed as another auxiliary subunit of Ca_V_1.1 and it represents the fourth essential component of skeletal muscle EC coupling. In fact, RyR1, Ca_V_1.1, β_1a_, and STAC3 (plus junctophilin2, which is important for the formation of plasma membrane-SR junctions but not essential for EC coupling) were sufficient to reconstruct skeletal muscle-like depolarization-induced calcium release in heterologous cells [69], indicating that these four proteins represent the full complement of essential EC coupling proteins. Considering the status of STAC3 as a factual auxiliary subunit of Ca_V_1.1 channels and its essential role in skeletal muscle EC coupling, it too needs to be included in the consideration of Ca_V_1.1-related channelopathies. In fact, a mutation in STAC3 causes a rare form of myopathy initially described in Native Americans, and therefore named Native American myopathy (NAM) [36].

## Calcium channel function of Ca_V_1.1 in skeletal muscle

So far, we considered that Ca_V_1.1 and its associated proteins are essential for skeletal muscle EC coupling independently of Ca_V_1.1’s role as calcium channel and therefore that perturbed Ca_V_1.1 function inevitably will result in EC coupling pathology but not in a channelopathy in the classical sense. This is impressively demonstrated in mouse models in which the channel function of Ca_V_1.1 has been perturbed or occluded without compromising its function as voltage sensor for EC coupling [19, 48]. These mice lack skeletal muscle L-type calcium currents but show no muscle pathology whatsoever. Therefore, Ca_V_1.1 L-type calcium currents are dispensable for normal growth and function of skeletal muscle. Consequently, calcium channel loss-of-function mutations in Ca_V_1.1 are not expected to result in disease. Does this imply that Ca_V_1.1 currents are irrelevant for disease? By no means. Apparently, it is important for normal muscle function to abolish or curtail the current function [24]. Consequently, any gain of channel function might have pathological effects.

Before we will turn to such diseases, let us briefly consider how and when calcium currents are essentially abolished under physiological conditions in skeletal muscle. Of all the voltage-gated calcium channels, the classical skeletal muscle L-type calcium currents are the slowest in activation, the ones with the most right-shifted voltage dependence of activation, and they exhibit small current amplitude. Therefore, the 1–2 ms depolarization of a skeletal muscle action potential will hardly activate opening of this channel, not even when delivered as high-frequency bursts. The action potential barely reaches the voltages necessary for Ca_V_1.1 activation, and if so, within this brief pulse, the slow activation kinetics will not allow a substantial response. Finally, should a small number of channels still activate under these unfavorable conditions, these channels are characterized by an extremely low open probability. Interestingly, in mammalian Ca_V_1.1 channels, a multitude of mechanisms contribute to this downregulation of calcium currents. The slow activation kinetics is encoded in the specific amino acid sequence of the voltage-sensing domain (VSD) of the first repeat [62, 97]. The right-shifted voltage dependence of activation and the low open probability are determined by structures in the VSD of the fourth repeat [96, 97]. Specifically, the insertion of an alternatively spliced exon into the extracellular linker of transmembrane helices VIS3 and VIS4 causes a 30-mV right shift of V½ and a > 5-fold reduction of current density (Fig. 1b–d). Importantly, the activation of channel opening is uncoupled from activation of EC coupling in that depolarization-induced calcium release is activated with fast kinetics and at 30 mV less depolarized potentials relative to current activation. Accordingly, inclusion of the 19 amino acids encoded by exon 29 into the IVS3-S4 linker right-shifts the voltage dependence of current activation, but not of EC coupling [96]. Finally, the auxiliary α_2_δ-1 subunit further slows down activation kinetics [68]. Interestingly, this effect of α_2_δ-1 is specific to Ca_V_1.1, as with Ca_V_1.2 the α_2_δ-1 subunit has exactly the opposite effect [95]. Altogether, having four distinct mechanisms at work to curtail Ca_V_1.1 currents suggests that in mature skeletal muscle L-type calcium currents are not only dispensable but also probably disadvantageous.

If this is the case, why not abolish calcium conductance completely? Actually, teleost fish utilize this strategy by expressing non-conducting Ca_V_1.1 channels [79]. Perhaps, in mammals, Ca_V_1.1 currents are needed in a different context. Indeed, the predominant splice variant in embryonic skeletal muscle lacks exon 29 and thus has substantially different current properties [85, 94]. The embryonic Ca_V_1.1e channel variant has a voltage dependence of activation and current density comparable to the cardiac/neuronal Ca_V_1.2 (Fig. 1d). In other words, during development, skeletal muscle expresses a normal calcium channel allowing calcium entry in response to spontaneous and motor nerve-induced electrical activity. Nevertheless, if depolarization-induced calcium influx in developing muscle cells were essential, loss-of-function mutations affecting current properties would be expected to result in developmental pathology of the motor system. At least in mice, this is highly unlikely, because mice expressing non-conducting Ca_V_1.1 do not display muscle pathology [19]. This does, however, not exclude the possibility that during early development the calcium-conducting Ca_V_1.1e is involved in physiologically relevant functions. For example, recently, we and others discovered a crucial role of Ca_V_1.1-dependent calcium signals in the earliest stages of neuromuscular junction development [15, 43]. Patterning of postsynaptic acetylcholine receptors in the synaptic target zone in the center of the muscle fibers was highly sensitive to the size of the muscle calcium signals. Importantly, in the absence of RyR1, calcium influx through the Ca_V_1.1e channel was sufficient for normal neuromuscular junction development, indicative of its physiological role during development. Although calcium influx was not essential, as in mice with non-conducting channels the subsequent onset of EC coupling compensated for the loss of channel function [43].

Whereas loss of Ca_V_1.1 channel function is inconsequential, would a gain of channel function be harmful? The observation that curtailing calcium currents is a process actively regulated during development in itself indicates that calcium influx during normal EC coupling might be disadvantageous. Indeed, a mouse model in which the developmentally regulated inclusion of exon 29 (resulting in the poorly conducting Ca_V_1.1a) was occluded showed severe effects on muscle differentiation and health [85]. First, the extra calcium influx caused an aberrant fiber type specification. Both in predominantly fast extensor digitorum longus (EDL) and in slow soleus muscle, the fiber type composition was substantially shifted towards slower myosin isoforms, with the expected effects on muscle strength and fatigability. Furthermore, with increasing age, the continuing calcium influx caused severe damage and loss of mitochondria, resembling a disease phenotype characteristic for mouse models with increased calcium load in skeletal muscles [6]. Thus, even though the channel function of Ca_V_1.1 is dispensable for normal skeletal muscle physiology, it is still possible that gain-of-function mutations causing calcium influx in adult muscle will give rise to a Ca_V_1.1 channelopathy.

## Potential Ca_V_1.1-related pathologies in tissues other than skeletal muscle

Now let us briefly consider the possibility that Ca_V_1.1 channels might contribute to body functions in other tissues and that genetic variants resulting in loss or gain of function might cause non-muscle Ca_V_1.1 channelopathies. Using whole exome sequencing, a missense mutation *CACNA1S* (I289V) has been linked to aberrant tooth morphogenesis in several individuals of 5 Thai families [47]; however, the mechanistic link to a Ca_V_1.1 function has not been explored. Several reports indicate the expression of Ca_V_1.1 in activated T-lymphocytes [3] where it contributes to the calcium signal in response to T cell receptor stimulation. Intriguingly, this channel lacks exon 29 [55], suggesting that it may possess the improved gating properties of the splice variant expressed in embryonic skeletal muscle. Nevertheless, how this voltage-dependent channel is activated upon T cell receptor activation in non-excitable cells remains a mystery. Several studies show immunostaining of Ca_V_1.1 in synapses of retinal bipolar cells [82, 98]. However, confirmation of Ca_V_1.1 expression with independent methods and a characterization of its possible role in retinal function are still lacking and indeed this finding may reflect antibody cross-reactivity [34]. At the time of this publication, Ca_V_1.1 channelopathies with deficiencies in the immune system or vision have not been reported, indicative of merely non-essential functions of Ca_V_1.1 channels in these tissues. This notion is further supported by the observations that the mouse models expressing non-conducting Ca_V_1.1 (loss-of-function) [19] or Ca_V_1.1 excluding exon 29 (gain-of-function) [85] did not reveal deficiencies in vision nor in the immune response. Thus, for the time being, Ca_V_1.1 channelopathies principally represent skeletal muscle diseases.

## Skeletal muscle channelopathies

**Fig. 2:**
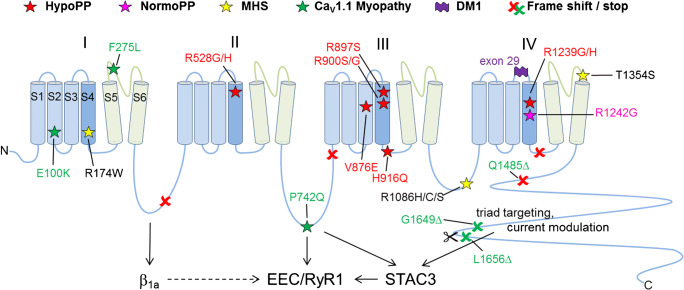
Positions of disease mutations in the domain topology model of Ca_V_1.1. *CACNA1S* variants and splicing defects linked to muscle disease primarily concern functionally important residues of the VSDs and of the P-loop involved in ion conduction and selectivity. In addition, substitutions and truncations of intracellular loop sequences directly or indirectly affect interactions with associated proteins. Accordingly, some of the mutations exert their effects by directly altering the properties of Ca_V_1.1 (calcium currents, omega currents), whereas other mutations cause disease by altering the interactions with, or the function of, the RyR1 (EC coupling, calcium leak). Cylinders S1–S4 shaded in blue represent the voltage-sensing domains (VSDs), and cylinders S5–S6 shaded in light green represent the pore domains (PDs) of repeats I, II, III, and IV. HypoPP, hypokalemic periodic paralysis; NormoPP, normokalemic periodic paralysis; MHS, malignant hyperthermia susceptibility; myotonic dystrophy type 1, DM1. Red *X*, truncations probably resulting in non-functional channel fragments; green *X*, truncations compatible with functional expression of the channel

Clinically, skeletal muscle channelopathies manifest as recurring episodes of muscle weakness or muscle stiffness triggered by exercise, cold stress, excessive potassium uptake, or volatile anesthetics. Episodic muscle weakness (periodic paralysis) is caused by the transiently reduced excitability of the muscle. Usually, it comes in two forms distinguished by the potassium levels during an attack: hypokalemic periodic paralysis (HypoPP) and normokalemic periodic paralysis (NormoPP). Muscle stiffness, called myotonia, is caused by uncontrolled repetitive firing of action potentials. Malignant hyperthermia (MH) susceptibility represents another channelopathy affecting skeletal muscle. Typically, it occurs as a crisis during application of volatile anesthetics or depolarizing muscle relaxants and manifests as an attack of extreme muscle contractures accompanied by increased metabolism, increased body temperature (therefore its name), and damage to the musculature. Congenital myopathies represent a clinically and genetically heterogenous group of early-onset muscle diseases characterized by pronounced muscle weakness and distinctive histological abnormalities. Myotonic dystrophy combines the symptoms of transient hyperexcitability and chronic muscle wasting. For all these muscle diseases, an involvement of ion channel genes including *CACNA1S* has been demonstrated (Fig. 2). In addition, recently, a rare but debilitating muscle disease (Native American myopathy, NAM) has been linked to mutations in STAC3, a skeletal muscle-specific scaffolding protein intimately linked to the function of Ca_V_1.1 in EC coupling.

## Hypokalemic periodic paralysis (HypoPP)

HypoPP is a dominantly inherited autosomal disease characterized by episodes of flaccid generalized muscle weakness accompanied by low serum potassium levels (< 3.5 mM) [10]. In the majority of patients, HypoPP is accompanied by permanent progressive muscle weakness and muscle degeneration. Episodes of muscle weakness are triggered by rest after exercise, hypokalemia following intake of carbohydrates or insulin administration, and cold stress. During an attack, voltage-gated sodium channels become inactivated by long-lasting membrane depolarization (from − 90 to − 60 mV), which, paradoxically, is associated with a reduction in extracellular potassium concentrations [42]. The genetic cause of HypoPP is mutations in two voltage-gated cation channels, Ca_V_1.1 (in approximately 60% of the cases) and Na_V_1.4 (in approximately 20%). Importantly, almost all known causative mutations in both channels neutralize gating charges of the S4 helices of the voltage-sensing domains (VSDs) (Fig. 2). These positive gating charges (usually between 4 and 6 arginines and lysines in every third position of S4) serve multiple important functions in the voltage-sensing process. They are the positive charges attracted by the force of the transmembrane electric potential, thus pulling the S4 helix inward and outward at rest and depolarization, respectively. They form transient ion-pair interactions with negative countercharges in other parts of the VSD to facilitate the state transitions and to stabilize the resting and activated states. And the sequential positioning of gating charges in the hydrophobic constriction site (HCS) seals the otherwise hydrophilic gating pore through which S4 helix slides upon activation and deactivation. Therefore, it is highly plausible that neutralizing mutations of the gating charges will cause defects in channel gating, and initially, such defects were suspected to be the cause of HypoPP.

**Fig. 3:**
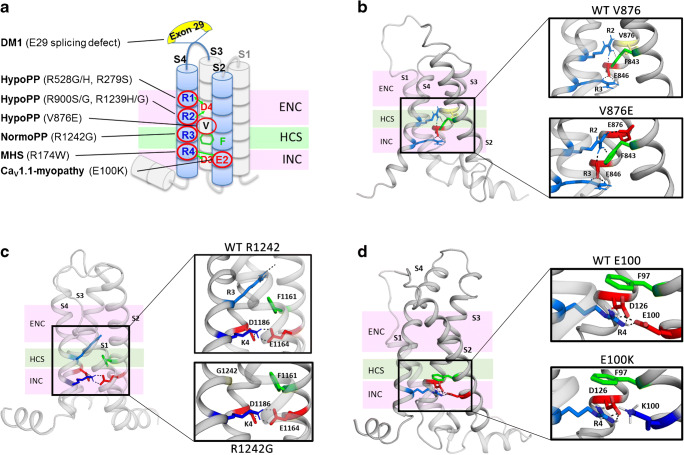
Ca_V_1.1 voltage-sensing domains are hot spots for disease mutations. **a** Schematic drawing of a generic VSD with the positions of currently known *CACN1S* disease mutations. Charge neutralizations of the gating charges R1 to R3 in the S4 helix cause state-dependent omega leak currents leading to HypoPP or NormoPP, respectively. Adding a negative charge (V876E) in the neighboring S3 helix may have the same effect. Changing the charge of the innermost gating charge R4 or its ion-pair partner E2 perturbs the function of the VSD. **b** The structure model of a VSD shows how specifically in the resting state the extra negative charge in the non-canonical HypoPP mutant V876E allows the gating charge R2 to form an additional ion pair above the hydrophobic constriction site (HCS). **c** The NormoPP mutation R1242G removes the gating charge R3, which is positioned above the HCS in the activated state and just below in the resting state, consistent with the reported state-dependent bi-directional omega currents. **d** The loss of a counter charge in the myopathy mutant E100K weakens the stabilizing ion-pair interactions of the gating charge located just below the HCS. Structure models, courtesy of M. Fernandez-Quintero

The well-known HypoPP mutations in Ca_V_1.1 are R528G/H, the outermost arginine (R1) in the VSD of the second repeat (VSD II); R897S and R900S/G, corresponding to R1 and R2 in VSD III; and R1239H/G, corresponding to R2 in VSD IV (Figs. 2 and 3). Early biophysical characterization of calcium current properties of Ca_V_1.1(R528H/G) and Ca_V_1.1(R1239H/G) in heterologous cells and myotubes indicated a loss of channel function [40, 46, 60, 61]. The mutant channels showed slowed activation, a reduction of open probability and current amplitude, and a left-shift in the voltage dependence of the steady-state inactivation. These current defects were accompanied by action potential broadening and a reduction of its amplitude. However, none of these changes in the current properties of the channel variants could explain the long-lasting depolarizations observed in HypoPP muscles. Let alone the fact that L-type calcium currents are dispensable for normal muscle function, and therefore loss of channel function hardly could cause muscle disease (see above). Similarly, the loss-of-function effects of HypoPP mutations observed in Na_V_1.4 were equally inconsistent with the observed depolarization phenotype. So what do these mutations in Ca_V_1.1 and Na_V_1.4 have in common that could cause long-lasting membrane depolarizations and HypoPP?

The answer is “omega currents.” Omega currents (also called gating pore currents) are leak currents through VSDs of voltage-gated cation channels with mutations of one of the gating charges [14]. Gating pore currents are unrelated to the ionic currents through the channel’s pore domain (PD) and much smaller than these (< 1%). The anomalous ion conduction pathway is established by the misalignment of the mutated gating charge (R1 or R2 in Ca_V_1.1) and the hydrophobic constriction site (HCS), thus abolishing the seal between the outer hydrophilic vestibule and the cytoplasmic compartment [59]. Omega currents are state-dependent in that, depending on the position of the mutated gating charge along the transmembrane S4 helix, they typically occur at hyperpolarized (resting) or depolarized (activated) conditions. In the case of R1 or R2 mutations, the omega pore opens at hyperpolarized potentials when these gating charges reside in the HCS. In contrast, depolarization pulls the S4 helix outward so that the intact inner gating charges occupy the HCS and seal the leak. Contingent on the channel type and the nature of the amino acid substitution (bulky or small), omega pores conduct protons and/or monovalent cations. Today, such leak currents are generally accepted to be the initial cause of the long-lasting membrane depolarization observed in HypoPP muscle fibers.

Omega currents were first described in voltage-gated potassium channels [83] and later identified as the cause of hyperkalemic and normokalemic periodic paralysis in Na_V_1.4 mutants [80, 84]. For the longest time, direct biophysical identification and characterization of omega currents in the Ca_V_1.1 HypoPP mutants were hampered by the inability to functionally express Ca_V_1.1 channels in non-muscle expression systems. However, the observation that muscle fibers from patients and mice carrying the R528H mutation exhibit inward cation leak currents at hyperpolarized potentials with omega current characteristics strongly supported the notion that omega currents underlie HypoPP in patients with *CACNA1S* mutations [41, 104]. More recently, expression of a typical (R1239H) and an atypical (V876E) HyppoPP mutant in mouse muscle demonstrated omega pore currents carried by protons and sodium leak, respectively [29, 30]. Finally, a recent advance in heterologous expression of Ca_V_1.1 by co-expressing STAC3 made it possible to directly record and characterize the omega leak currents in the Ca_V_1.1-R528H and -R528G HypoPP mutants in the oocyte expression system [107]. The improved recording conditions compared to recordings in muscle fibers or myotubes allowed the analysis of the permeation properties of the two variants. Surprisingly, the leak current in Ca_V_1.1-R528H (the most frequent HypoPP mutation) was carried primarily by sodium and not by protons as is the case in the corresponding mutations in Na_V_ and K_V_ channels. Together, these experiments unambiguously establish omega pore leak current as the patho-mechanism of Ca_V_1.1-linked HypoPP. Furthermore, the new expression system now affords the opportunity to systematically characterize all known Ca_V_1.1 HypoPP variants.

How does this omega current lead to the long-lasting depolarizations during attacks of HypoPP and why is it triggered by low external potassium concentrations? Remarkably, skeletal muscle cells possess a bistable resting potential, with one stable state near the K^+^-equilibrium potential (at − 80 mV) and another at about − 50 to − 60 mV [42]. The latter arises from the balance of an outward current through an inward rectifying K^+^ channel and a linear inward leak current. Therefore, both lowering the extracellular potassium concentration and/or increasing the inward leak current can shift the membrane potential into the less polarized stable state. Both these conditions occur during attacks in muscles expressing HypoPP mutant Ca_V_1.1 channels. Shifting the membrane potential into the second stable state causes the inactivation of Na_V_1.4 channels and the temporary inexcitability of the muscle cells.

In the long run, HypoPP patients frequently suffer from muscle edema and chronic weakness due to cytoplasmic sodium overload. Both the acute attacks of muscle weakness and the chronic defects associated with edema could be effectively rectified by treatment with repolarizing drugs, like carbonic anhydrase inhibitors, that shift the muscle membrane potential from the depolarized state (P2) back to the resting (P1) state [41]. Targeting serum potassium levels by treatment with aldosterone antagonists is another effective treatment option showing greatly promising results in HypoPP patients [101]. This drug effect may at least in part be due to the stimulation of the Na,K-ATPase activity, which counteracts the accumulation of sodium in the cytoplasm and of potassium in the extracellular space, and repolarizes the muscle membrane [7]. Thus, understanding the molecular pathology of the Ca_V_1.1 HypoPP mutations and in particular identifying omega currents as its primary cause greatly facilitated the development of effective therapies.

Interestingly, a non-canonical *CACN1S* variant has been described in a four-generation South American family with severe HypoPP [44]. V876E is positioned in the S3 helix of VSD III. It is unusual in that it does not concern a gating charge in the S4 helix but adds a negative charge in an adjacent helix. Whether this too results in omega currents and, if so, by which molecular mechanism has not been studied. One possibility is that in the resting state the extra negative charge in the S3 helix attracts a positive gating charge and thereby rearranges the HCS in a manner that a hydrophilic conduction pathway for omega currents is formed (Fig. 3b). Another variant H916Q in a family with complete penetrance of HypoPP in males but not in females has been described [49]. H916 is a conserved residue in the IIIS4-S5 linker that connects the VSD with the PD and is known to be important for channel gating, although the potential disease mechanism is still elusive.

In rare cases, HypoPP has been associated with motor neuron degeneration and progressive muscle atrophy. Many cases of progressive muscle atrophy are clinically and pathologically linked to amyotrophic lateral sclerosis. However, progressive muscle atrophy represents a complex genetic disorder in which for most patients the genetic cause is unknown. In one such family, HypoPP has been linked to the known R528H variant in Ca_V_1.1, suggesting the possibility that the *CACNA1S* gene may represent a genetic risk factor for progressive muscle atrophy [57].

## Normokalemic periodic paralysis (NormoPP)

As described above, omega currents are state-dependent contingent on which gating charge is mutated. While the HypoPP mutations in Ca_V_1.1 affect the two outer arginines (R1 and R2), in Na_V_ channels also, disease-causing mutations of R3 have been described [81]. Channels with this mutation typically conduct omega currents in the activated state and lead to muscle weakness associated with normal potassium levels. For Ca_V_1.1, a family with a mutation of R3 in the fourth VSD (R1242G) has been described that presented with complex muscle pathology associated with normokalemic periodic paralysis [22]. When expressed in dysgenic myotubes, this Ca_V_1.1 variant displayed a reduced current amplitude and a left-shifted voltage dependence of steady-state inactivation resulting in a decreased slope and amplitude of the muscle action potential. These changes of the macroscopic current properties may be direct effects of the loss of the R3 gating charge or result from an outward omega current observed at positive membrane potentials. Interestingly, also an inward omega current has been observed at negative potentials after long depolarizations. Thus, depending on the position of the mutated (lacking) gating charge (R3) relative to the HCS—below it at rest and above it during activation—this mutation may cause state-dependent bi-directional omega currents (Fig. 3c). These leak currents cause muscle weakness due to the loss of excitability by inactivating Na_V_1.4 and at the same time trigger continuous depolarization-induced calcium release, consistent with the complex myopathy symptoms. It would be of great interest, both for understanding the exact patho-mechanism for R2142G and for better mechanistic understanding of Ca_V_1.1 voltage-sensor function, if the biophysical properties of this disease mutation would be further analyzed in the new oocyte expression system.

### Ca_V_1.1 splicing defects—myotonic dystrophy type 1 (DM1)

Myotonic dystrophies (DM1 and DM2) are autosomal dominant disorders characterized by symptoms in multiple organs including skeletal myopathy, specifically muscle weakness and slow muscle relaxation called myotonia. Genetically, both DM1 and DM2 are characterized by tandem repeat expansions in non-coding regions of the genome [91]. DM1 is caused by CTG repeats in the 3′ untranslated region of the dystrophia myotonica protein kinase, and DM2 by expanded CCTG repeats in cellular nucleic acid binding protein. The toxic expanded CUG or CCUG RNAs bind and sequester the splicing factor muscleblind-like 1 [50]. This in turn causes the misregulated splicing of a multitude of proteins including the CLCN1 chloride channel, the insulin receptor, and the skeletal muscle calcium channel Ca_V_1.1. Myotonia is likely caused by the loss of CLCN1 function, which triggers involuntary runs of muscle action potentials [52, 53]. In addition, a gain of Ca_V_1.1 channel function likely contributes to muscle dystrophy in MD1 patients.

The splicing defect in Ca_V_1.1 concerns the inclusion of exon 29 [89]. During normal development, embryonic Ca_V_1.1e channels, lacking exon 29, are completely replaced by the adult Ca_V_1.1a channel variant, containing exon 29 [85, 96]. Inclusion of exon 29 dramatically right-shifts the voltage dependence of activation by 30 mV and reduces the current amplitude greater than fivefold. Thus, alternative splicing of exon 29 renders the skeletal muscle calcium current small and less responsive to depolarization, while fully maintaining its activity as the voltage sensor of EC coupling. In DM1 patients, this splicing event is reversed, leading to the aberrant expression of the embryonic Ca_V_1.1e splice variant in adults. Noticeably, the fraction of embryonic Ca_V_1.1e expressed in DM1 patients correlated with the degree of clinically assessed muscle weakness [89]. Thus, it is expected that DM1 patient skeletal muscles with every movement experience a pathologically enhanced calcium influx.

Likewise, in muscles of a DM1 mouse model, the additional knockdown of muscle blind resulted in the upregulation of Ca_V_1.1e and aggravated muscle pathology evidenced by an increased frequency of centrally located myonuclei [89]. Thus, missplicing of Ca_V_1.1 exon 29 contributes to the DM1 pathology in mice. On the other hand, a mouse model in which insertion of exon 29 had been abolished and therefore the embryonic Ca_V_1.1e splice variant is exclusively expressed throughout life did not display DM-like muscle weakness [85]. This is consistent with the notion that myotonia primarily arises from the deficiency in chloride channel function. However, progressively, the muscles of this mouse model showed mitochondrial damage and ultimately a severe loss of mitochondria that was accompanied by reduced endurance [85]. Such a phenotype is commonly observed in muscular dystrophy mouse models, the muscles of which experience chronic calcium overload, thus supporting the conclusion that the increased calcium influx in muscles aberrantly expressing the embryonic Ca_V_1.1e splice variant causes myopathy. In human DM1 patients, these slowly progressing defects may be exacerbated by the combined defects of multiple misspliced genes as well as with increasing age. In conclusion, the gain of the Ca_V_1.1 channel function likely contributes to the pathology of DM1.

Currently, no disease-modifying treatments for DM1 and DM2 exist and therapy is limited to symptomatic treatments and preserving motor function. As DM involves multiple misspliced and dysregulated proteins, the affected ion channels, CLCCN1 and Ca_V_1.1, are no viable targets. Rather the most promising experimental treatment strategies target the common upstream cause the toxic expanded CUG or CCUG RNAs [91].

### Malignant hyperthermia susceptibility

MH susceptibility is an autosomal dominantly transmitted predisposition to respond with uncontrollable calcium release and consequently massive muscle contractions to volatile anesthetics and depolarizing muscle relaxants [20]. The symptoms of an MH reaction include muscle rigidity, acidosis, rapidly raising body temperature and usually lead to muscle breakdown and the death of the patient if left untreated. Otherwise individuals affected by this pharmaco-genetic condition are clinically inconspicuous. In the majority of MH susceptible individuals (> 70%) the massive calcium release is linked to functional missense mutations within the RyR1 release channel itself [93]. Close to 200 such mutations have been identified in all parts of the RyR1. The commonality of MHS mutations in RyR1 is that these destabilize the closed state of the calcium release channel and make it hypersensitive to activation by triggering agents like volatile anesthetics or caffeine. MS susceptibility mutations also cause leaky RyR1 channels, depletion of the sarcoplasmic reticulum calcium stores and chronically elevated myoplasmic calcium concentrations at rest. These symptoms are frequently associated with a myopathy called central core disease (CCD), because of an abundance of central cores in type 1 muscle fibers, and clinically manifests as congenital muscle hypotonia with delayed motor development [39].

In addition to the MHS mutations in RyR1, several MH susceptibility mutations (representing about 1% of the genetically solved cases) have been identified in the *CACNA1S* gene [11, 21, 58, 70, 92]. The R1086H/C/S mutations affect a highly conserved arginine residue located in the cytoplasmic loop connecting repeats III and IV of Ca_V_1.1 (Fig. 2). Biophysical analysis in *dysgenic* myotubes reconstituted with Ca_V_1.1 carrying the R1086H substitution demonstrated that this mutation somewhat reduced the current density and increased the sensitivity of calcium release to activation by depolarization and by caffeine [102]. The T1354S MHS mutation located in the outer pore region of Ca_V_1.1 accelerated activation kinetics of the L-type calcium current and again left-shifted the sensitivity of calcium release to caffeine [70]. Evidently, this mutation in the voltage sensor of EC coupling (Ca_V_1.1) caused the hypersensitivity of the calcium release channel (RyR1) to physiological and pharmacological activation, and thus functionally mimicked the disease-causing effects of mutations in the RyR1 itself. Interestingly, the mutation in the third MHS site in Ca_V_1.1, R174W, affects the innermost gating charge of the first VSD and displayed somewhat different effects. Unlike the other Ca_V_1.1 MHS mutations, R174W did not alter EC coupling but essentially ablated the L-type calcium currents [21]. Nevertheless, the sensitivity of calcium release to caffeine and volatile anesthetics was increased, sarcoplasmic reticulum calcium stores were partially depleted, and conversely, the resting myoplasmic calcium concentration was increased. These differential effects indicated that the R174W mutation stabilized the affected VSD I of Ca_V_1.1 in the resting state, ablating current activation, and independently interfered with the ability of Ca_V_1.1 to stabilize the RyR1 in a closed conformation, resulting in a leaky calcium release channel. While the loss of channel function may be inconsequential for normal muscle function and the health of affected patients, the chain of events initiated by the leaky RyR1 probably gives rise to the hypersensitivity of the calcium release channel characteristic of MHS. Notably, neither of the two defects in the function of Ca_V_1.1 affected its ability to activate EC coupling. The molecular mechanism by which these three HMS mutations in structurally and functionally distinct domains of Ca_V_1.1 sensitize RyR1 calcium release to activation by caffeine and volatile anesthetics is unknown. The affected Ca_V_1.1 domains have not been implicated in interactions with the RyR1 or in a functional role in EC coupling. Nevertheless, their common effect on RyR1 function and pharmacology impressively demonstrates the complex interactions between the voltage sensor and the effector release channel within the macromolecular EC coupling apparatus.

As MH crises are mostly limited to the clinical setting, therapeutic efforts concentrate on their avoidance by testing patients potentially at risk either functionally, with a caffeine-sensitivity test, or genetically for known disease-causing mutations. MH crisis management is accomplished by whole-body cooling and the rapid administration of the MH antidote dantrolene [51].

### Ca_V_1.1-related myopathy

Congenital myopathies represent a genetically heterogenous group of early-onset, non-dystrophic muscle diseases characterized by varying degrees of muscle weakness and distinctive histopathological abnormalities [39]. The severity of muscle dysfunctions ranges from severe fetal akinesia to milder forms of hypotonia and muscle weakness. The disease-characterizing histopathological features include central cores, multi-minicores, central nuclei, and nemaline rods. Congenital myopathies are mostly disorders of EC coupling and altered calcium handling, and numerous mutations in the RyR1 gene have been identified as the cause of myopathy [93]. Therefore, it was not unexpected that recent whole exon sequencing studies also identified several (altogether 12) putative myopathy mutations in the *CACNA1S* gene in families presenting with perinatal hypotonia, severe axial and generalized weakness, and, in several cases, ophthalmoplegia [38, 76]. Genetically, the identified cases include compound recessive mutations, dominant mutations, and de novo mutations in various domains of Ca_V_1.1. All recessive cases described in these studies carried at least one nonsense mutation causing a frame shift resulting in the premature stop and consequently the dysfunction and/or loss of the Ca_V_1.1 protein. Although at present functional characterizations of the missense myopathy mutations are lacking, their positions in domains with known functions allow prediction as to the effects of the mutations and to possible patho-mechanisms.

For example, the recessive missense mutation E100K replaces a highly conserved negative counter charge within the charge transfer center of the first VSD with a positively charged residue. According to the sliding helix model of voltage sensing, this residue is critical for the translocation of the S4 helix through the membrane electrical field upon depolarization [14]. The reversal of the charge in the E100K mutant will prohibit the sequential formation of ion pairs between the gating charges and E100, and thus is expected to severely impede voltage sensing and activation of L-type calcium currents. Yet, this defect is not necessarily expected to interfere with EC coupling, which is likely independent of a functional first VSD [24]. Remember that also the MHS mutation R174W, representing the corresponding gating charge of VSD I, ablated calcium currents but not EC coupling (see above) [21]. In contrast, the dominant de novo missense mutation P742Q resides in the sequence of the cytoplasmic loop between repeats II and III that is critical for skeletal muscle-specific EC coupling. Previously we demonstrated that substitution of this residue with threonine (P742T), the residue found in the corresponding position of Ca_V_1.2, diminishes depolarization-induced calcium release by the RyR1 without affecting Ca_V_1.1 calcium currents [45]. Therefore, the disease mutation in this position is expected to specifically perturb the functionally important interaction of Ca_V_1.1 with the RyR1, and possibly with STAC3 [103], and thus obstruct EC coupling. Together, these two missense mutations suggest the intriguing possibility that mutations expected to specifically perturb either the channel function or the EC coupling function of Ca_V_1.1 both result in a similar disease phenotype. This is unexpected in light of the evidence demonstrating that L-type calcium currents are expandable for normal development and function of skeletal muscles in mice [19].

Another missense mutation, F275L, affects a conserved phenylalanine in the extracellular loop between S5 and S6 of the first repeat. This P-loop contributes to the channel ion selectivity filter [106] although the mutated residue is not part of the selectivity filter proper. Nevertheless, the mutation of a conserved residue in this domain may compromise the calcium influx through the channel pore. Again, this raises the question as to the possible role of Ca_V_1.1 calcium currents in causing muscle disease. However, also the MHS mutation T1354S in the P-loop of the fourth repeat altered channel gating and caused MHS (see above) [70]. Finally, several of the Ca_V_1.1 myopathy frame shift mutations cause truncation of the C-terminus of Ca_V_1.1 that still might allow expression of functional channels. Overall, the C-terminus of Ca_V_1.1 can be divided into two sections: a structurally conserved proximal part, containing multiple calcium/calmodulin regulatory elements, an interaction site for STAC3, and the triad targeting signal [26, 65, 103]. And the non-conserved distal C-terminus of Ca_V_1.1 that can be cleaved without affecting EC coupling or L-type calcium currents [25, 26]. Nonetheless, this distal fragment contains functionally relevant phosphorylation sites and regulatory domains and in vivo may remain attached to the channel complex [37, 54]. The nonsense mutation Q1485 resulting in truncation in the middle of the proximal C-terminus preserves the two EF hands but excludes the sequences important for triad targeting and interaction with STAC3. Most likely these channel variants, if expressed at all, fail to correctly incorporate into the junctional EC coupling apparatus and therefore will not support EC coupling. The recessive frame shift mutants at residues G1649 and L1656 truncate the C-terminus of Ca_V_1.1 close to its intrinsic cleavage site. Channels truncated near this site (at position 1661) are normally targeted into skeletal muscle triads and fully support calcium currents and EC coupling. Nevertheless, in myotubes derived from biopsies of a patient carrying the heterozygous L1656R mutation depolarization-induced calcium transients were substantially reduced [76]. Further functional analysis of these newly identified disease mutations will be necessary to fully understand how the specific amino acid substitutions in Ca_V_1.1 might cause congenital myopathy and in order to develop mechanism-based therapeutic strategies. On the other hand, detailed biophysical analysis of these myopathy mutations may reveal the significance of hitherto unnoticed molecular domains in the dual function of Ca_V_1.1 as L-type calcium channel and voltage sensor of EC coupling in skeletal muscle.

### Native American myopathy (NAM)/Ca_V_1.1-associated proteins STAC3

The effects of the MHS-causing mutations in Ca_V_1.1 on caffeine-induced calcium release as well as the predicted effects of several congenital myopathy mutations on Ca_V_1.1-RyR1 coupling corroborate extensive physiological evidence showing the intimate interactions between Ca_V_1.1 and RyR1 within the EC coupling apparatus. Consequently, additional components of this macromolecular complex may similarly be disease causing and thus deserve consideration in the context of Ca_V_1.1 channelopathies. The skeletal muscle-specific Ca_V_ β_1a_ subunit and the muscle-specific scaffolding protein STAC3 are the other two essential EC coupling proteins. Whereas, at present, no β_1a_-associated diseases are known, mutations in STAC3 have recently been shown to cause a rare form of myopathy.

Native American myopathy (NAM) is an autosomal recessive disorder first described in Lumbee Indians carrying the homozygous missense variant W284S in the *STAC3* gene [36]. NAM causes a range of debilitating symptoms including muscle weakness, delayed motor development, and malignant hyperthermia susceptibility, which in an estimated one third of the patients lead to death before the age of eighteen. Meanwhile, the *STAC3* W284S variant has been found in myopathy patients with non-Native American ethnicity [33, 90] and novel *STAC3* variants were identified in patients presenting congenital myopathy symptoms and MHS; altogether constituting the category of STAC3-related congenital myopathies [108].

In skeletal muscle, the function of STAC3 is closely linked to the function of Ca_V_1.1, suggesting several possible pathways by which a defect in STAC3 function could interfere with the role of Ca_V_1.1 in EC coupling. STAC3 acts as a chaperone for functional membrane expression of Ca_V_1.1 in heterologous cells [71]. An interaction of STAC3 with the C-terminus of Ca_V_1.1 is important for its association with the channel in skeletal muscle triads [8], and, at least in the closely related Ca_V_1.2 channel isoform, this interaction involves the known calmodulin binding site and interferes with calcium-dependent inactivation of L-type calcium channels [9, 64, 72]. In addition, the SH3-1 domain of STAC3 interacts with the critical EC coupling domain in the cytoplasmic II–III loop of Ca_V_1.1 [73, 103]. Therefore, mutations interfering with any one of these functions are expected to result in altered EC coupling functions. Interestingly, as of today, all the verified and putative NAM mutations reside in or near the vicinity of the STAC3 SH3-1/Ca_V_1.1 II–III loop interface and weaken this interaction and consequently interfere with the functional coupling between the voltage sensor and the release channel [103]. Notably, this weakening of STAC3 SH3-1 binding to the Ca_V_1.1 II–III loop is not paralleled by altered expression in the triads or reduced STAC3-Ca_V_1.1 co-immunoprecipitation in preparations from patient skeletal muscle [108]. Together, the available evidence indicates that, although STAC3 interacts with Ca_V_1.1 via multiple interaction sites and also affects triad targeting and current properties of Ca_V_1.1, NAM mutations specifically interfere with Ca_V_1.1’s functional interaction with RyR1 involved in skeletal muscle EC coupling. While an altered communication between Ca_V_1.1 and RyR1 may also lead to leaky release channels, the precise mechanisms how reduced EC coupling causes the plethora of symptoms of NAM still remain to be elucidated.

## Conclusion

**Fig. 4:**
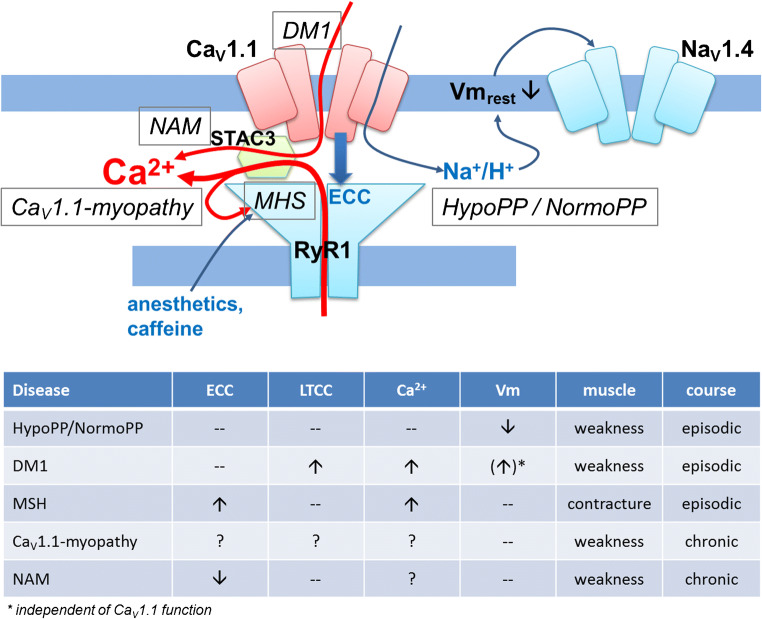
Multiple modes of altering muscle calcium handling and excitability cause Ca_V_1.1 channelopathies. Sodium and/or proton leak currents through mutated VSDs (omega currents; blue arrows at right) indirectly cause attacks of HypoPP or NormoPP, by inactivating voltage-gated sodium channels. An increased calcium load resulting from chronically increased influx (red arrows at left) contributes to chronic muscle wasting in DM1 and perhaps also in some Ca_V_1.1 myopathy mutations. Mutated Ca_V_1.1 can render the RyR1 hypersensitive to caffeine and anesthetics in MHS and possibly leaky in Ca_V_1.1-related myopathies. How loss-of-channel-function mutations in Ca_V_1.1 and mutations in Ca_V_1.1 and STAC3 that interfere with EC coupling cause myopathy remains unresolved to date

Together, this diverse catalog of Ca_V_1.1-related channelopathies reflects the dual physiological roles of Ca_V_1.1 as the voltage sensor of skeletal muscle EC coupling and, secondarily, as voltage-dependent calcium channel. With the exception of HypoPP and NormoPP, which were identified as gating-pore current diseases, the common cause of Ca_V_1.1-related channelopathies is altered calcium handling in skeletal muscle (Fig. 4). Gain-of-function mechanisms, causing either excessive calcium influx through misspliced Ca_V_1.1 or hypersensitive release of calcium by RyR1, are the likely causes of DM1 and MHS, respectively. A loss of the EC coupling interactions between Ca_V_1.1 and RyR1 appears to be the first step in the patho-mechanism of NAM. Whereas the variety of newly identified Ca_V_1.1 variants linked to congenital myopathies still await detailed functional characterization, they likely encompass the entire spectrum of gain- and loss-of-function mechanisms affecting both the channel and EC coupling function of Ca_V_1.1. The recent advances in molecular genetics have led to a rapid increase of the list of Ca_V_1.1-related diseases and will certainly improve the accurate diagnosis and targeted therapy of these diseases. Functional analysis of the molecular pathophysiology of the diseases-causing mutations substantially contributes to our understanding of the underlying physiological mechanisms and in some cases has shown the way towards more effective treatment strategies.
